# P-720. Are we aware of the RSV problematic in the Dominican Republic?

**DOI:** 10.1093/ofid/ofae631.916

**Published:** 2025-01-29

**Authors:** Rita A Rojas-Fermin, Anel E Guzman-Marte, Ann S Sanchez-Marmolejos, Francisco Guzman-Ricardo, Yeison Reyes, Ruben Calcano

**Affiliations:** Hospital General de la Plaza de la Salud, Santo Domingo, Distrito Nacional, Dominican Republic; Hospital General de la Plaza de la Salud, Santo Domingo, Distrito Nacional, Dominican Republic; Hospital General Plaza de la Salud, Santo Domingo, Distrito Nacional, Dominican Republic; Hospital General de la Plaza de la Salud, Santo Domingo, Distrito Nacional, Dominican Republic; Hospital General de la Plaza de la Salud, Santo Domingo, Distrito Nacional, Dominican Republic; SDI, Santo Domingo, Distrito Nacional, Dominican Republic

## Abstract

**Background:**

Respiratory Syncytial Virus (RSV) is a common cause of respiratory illness that could have a severe presentation in infants and elderly patients. As in other LATAM countries, RSV diagnosis is limited due to a lack of testing or access, and there is scarce data on the epidemiology, clinical impact, and mortality. Our aim was to characterize the disease in patients admitted to a hospital setting.

Distribution of admitted patients by age

**Figure:**
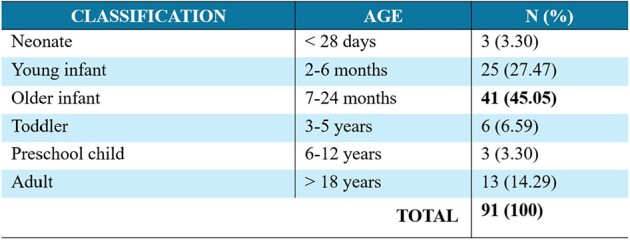

Source: Statistical registry of the authors

**Methods:**

We performed a retrospective review of admitted patients in a tertiary 289-bed hospital with a respiratory molecular panel from January 2022 to December 2023 (n = 715). RSV-positive patients (n = 91) were selected, and their clinical and demographic information, co-morbidities, risk factors, and outcomes were reviewed on their electronic medical record.

Microbial frequency in RSV coinfected patients

**Figure:**
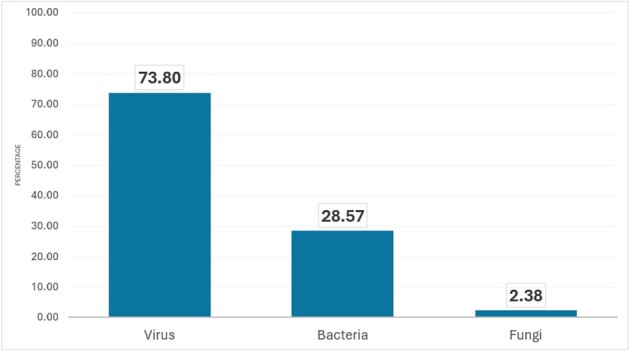

Source: Statistical registry of the authors

**Results:**

Out of 91 patients, 58.2% (53/91) were male, 75.8% were ≤2 years old, and 8.8% were ≥60 years old. The RSV positivity rate for all ages (0–84 years old) was 12.7% and 53.4% (69/129) for ≤2 years old. All cases were detected near the second semester of each year (May–December 2022 and July–December 2023).

Out of 50 patients with RSV as the only pathogen confirmed through testing, 32 developed respiratory complications (bronchiolitis and pneumonia), and 14% required critical care. Whereas 41 patients had the simultaneous presence of other pathogens, among them, 31 patients had ≥2 respiratory viruses detected.

The most frequent symptoms in RSV-only patients were cough (79.12%), dyspnea (67.3%), and wheezing (63.74%). Asthma was the most common respiratory comorbidity (16.48%), followed by low birth weight and preterm. The mortality rate was 4/91 (4.39%); among them were 2 patients < 6 months old with congenital cardiopathy and 2 elderly (69/78y) with Klebsiella pneumoniae-bacteremia.

Distribution of viral isolates from admitted patient respiratory and pneumonia panel samples.

**Figure:**
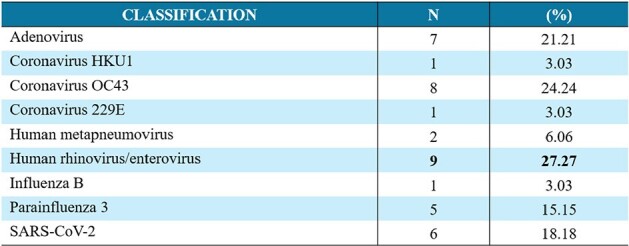

Source: Statistical registry of the authors

**Conclusion:**

RSV should be considered an all-ages respiratory infection, and it was found alone or in association with other respiratory pathogens mainly near the second semester each year. Studies like these are necessary in low-middle income countries like the Dominican Republic to address the burden of RSV and the impact that the vaccine applied during pregnancy and the elderly can have on disease prevention and complications. We encourage early and affordable diagnostic tests to curb the spread of RSV and reduce admissions.

**Disclosures:**

**Rita A. Rojas-Fermin, MD,FIDSA**, Gilead: Advisor/Consultant|Pfizer: Advisor/Consultant|Pfizer: Honoraria

